# Problematic Use of the Internet and Smartphones in University Students: 2006–2017

**DOI:** 10.3390/ijerph15030475

**Published:** 2018-03-08

**Authors:** Xavier Carbonell, Andrés Chamarro, Ursula Oberst, Beatriz Rodrigo, Mariona Prades

**Affiliations:** 1 FPCEE Blanquerna, Universitat Ramon Llull, 08022 Barcelona, Spain; ursulao@blanquerna.url.edu (U.O.); beatrizrc2@blanquerna.url.edu (B.R.); marionapo@blanquerna.url.edu (M.P.); 2 Departamento de Psicología, Universitat Autónoma de Barcelona, Cerdanyola del Vallès, 08193 Barcelona, Spain; andres.chamarro@uab.es; 3 Serra Hunter Program, Generalitat de Catalunya, 08029 Barcelona, Spain

**Keywords:** Internet addiction, mobile phone addiction, online social network, university students, technological addictions, behavioral addictions, CERI, CERM

## Abstract

It has been more than a decade since a concern about the addictive use of the Internet and mobile phones was first expressed, and its possible inclusion into the lists of mental disorders has recently become a popular topic of scientific discussion. Thus, it seems to be a fitting moment to investigate the prevalence of this issue over time. The aim of the present study was to analyze the prevalence of the perception of problematic Internet and smartphone use in young people over the period 2006–2017. To this end, a questionnaire on Internet use habits and two questionnaires on the negative consequences of Internet and smartphone use were administered to a sample of 792 university students. The scores were then compared with the results of former studies that had used these questionnaires. The perception of problematic Internet and mobile phone use has increased over the last decade, social networks are considered responsible for this increase, and females are perceived to be more affected than males. The current study shows how strong smartphone and Internet addiction and social media overlap. Participants from 2017 report higher negative consequences of both Internet and mobile phone use than those from 2006, but long-term observations show a decrease in problematic use after a sharp increase in 2013. We conclude that the diagnosis of technological addictions is influenced by both time and social and culture changes.

## 1. Introduction

Ever since Young [1] presented *Internet Addiction: the Emergence of a New Disorder* at the Congress of the American Psychological Association in Toronto, Internet addiction has been a widely discussed disorder in the media and in scientific literature [2]. The interest in the possible addiction to the Internet, video games, online role-playing games, television, and mobile phones has given rise to a new field of study: technological addictions [3]. In fact, the DSM-5 [4] included addiction to video games in the list of disorders that should receive further research. The negative consequences of this problem include the possible increase of stress, anxiety, and/or the “paradox” of a lack of communication, despite being more connected, especially among young people and adolescents [5,6,7].

Internet use and Internet access habits have recently evolved. For example, in 2015, in Spain, there was a clear preference for smartphones (88.2%) over computers (78.2%) for accessing the Internet, especially in 14–19 year olds. This evolution of preference for the phone over the computer was also observed for the accessing of leisure activities, which decreases the dominance of the computer in the professional and educational spheres [8]. Because of its popularity and because it is a relatively new device (compared to the “classical” mobile phone), the smartphone has raised concerns about its potential to be addictive [9,10,11,12] as happens with other possible behavioral addictions, such as those to the Internet or to social networking sites [13].

The prevalence of smartphone addiction has been established using different self-report measures. Among the most frequently used, and actually one of the first self-report questionnaires, there was the Mobile Phone Problem Usage Scale (MPPUS) [9,14], later translated and adapted to the Spanish population [15]. The Problematic Mobile Phone Use Questionnaire (PMPUQ) [16] has also been employed in several studies [17]. Another questionnaire used in the Spanish speaking context is the *Cuestionario de Experiencias Relacionadas con el Móvil* [Questionnaire for mobile phone-related experiences] (CERM) [18], used to collect data from young people [19] and university students [20,21]. Recently, several other questionnaires were adapted to asses smartphone addiction, like the Smartphone Addiction Scale (SAS) [22], the Smartphone Addiction Inventory (SPAI) [23], or the one used in Saudi Arabia [24]. All of them have shown their usefulness and good validity and reliability, but it is difficult to assess if problematic mobile phone use has increased over the years since researchers use different measurements and instruments.

The term ‘Internet addiction’ or ‘smartphone addiction’ is not used consistently in the literature. A review shows the use of different expressions; there is, for instance, ‘digital addiction’ [25], ‘problematic Internet use’ [26], or ‘Internet-use disorder’ [27]. Even the same authors use different expressions in different papers [27,28]. It is not the purpose of the present article to discuss the convenience of one term or another, nor to discuss if the negative consequences can be interpreted as an addiction or not; however, we will use the term ‘addiction’ for the studied phenomenon, because it was one of the first terms used [1] and because in the studies that we will compare, this term has also been used from the beginning.

Added to the scientific relevance of this issue, the media tend to echo and spread negative information about the use of mobile phones. Results of these alerts are concepts such as the so-called technostress [29], smombie (a combination of “smartphone” and “zombie”) [30], fear of missing out (“FoMO”) [31], and nomophobia (“no-mobile-phone phobia”) [32]. However, studies on the addictive consequences of both the ‘old’ mobile phone [9,33,34,35,36], the current smartphone [22,24,37,38,39,40], and the Internet are cross-sectional, and therefore the temporal evolution of their addictive impact on the population is still unknown. On the basis of the aforementioned research, the objectives of the current study were, first, to explore the perception of problematic Internet and smartphone use in young people in 2017 and, second, to compare these results with former those of studies that used the same measurement instruments, in order to analyze the evolution of the negative effects of Internet and mobile phone use over a period of ten years. As Internet and smartphone use has increased considerably during the past decade, we also expect negative effects to increase over the years (H1). As shown in the previous literature, also in our study women reported a greater use of social networking sites, whereas men used more videogames and adult pages (H2). We also expect women to experiment stronger negative consequences than males (H3).

## 2. Materials and Methods

### 2.1. Participants

In the present study, 792 students from Universitat Ramon Llull of Barcelona (Spain) participated in the study in May 2017. They were studying Psychology (30.7%), Physical Education and Sports Sciences (17.2%), Education (47.6%), and Speech Therapy (4.5%). The mean age was 21.6 years (SD = 3.3), and 76.5% were women.

The data on problematic Internet and mobile phone use obtained in the present study (called Cohort 6 hereinafter) was compared with data obtained from other cohorts of university students who answered the CERI and CERM questionnaires in studies conducted by our team between 2006 and 2017:

- Cohort 1: 322 students from the fields of Psychology, Physical Education, Nursing, Physiotherapy, or Communication in Universitat Ramon Llull of Barcelona. The mean age was 19.71 years (SD = 1.73), and 72.7% were women. The data were collected during the 2005–2006 academic year [18].
- Cohort 2: 318 Psychology students from the University of Illinois at Urbana-Champaign in the United States, 51% of which were women, aged between 17 and 21 years. The data were collected during the 2013–2014 academic year [20].
- Cohort 3: 425 Psychology students from the University of Illinois at Urbana-Champaign with an average age of 19.5 (SD = 1.5); 65.4% were women. The data were collected during the 2015–2016 academic year [21].
- Cohort 4: 308 Psychology students from the Universitat Ramon Llull. The mean age was 22.2 years (SD = 4.1), and 77.9% were women. The data were collected during the 2015–2016 academic year [21].
- Cohort 5: 308 psychology students from Ibagué University in Ibagué, Colombia. The mean age was 19.8 years (SD = 3.03), and 65.6% were women. The data were collected during the 2015–2016 academic year [21].

### 2.2. Instruments

The following instruments were used:

- Sociodemographic data and Internet use habits were assessed with an ad hoc questionnaire. This questionnaire collected sociodemographic data (age, sex, and university degree) and frequency and type of Internet use (e.g., gambling, social networks, etc.) in a five-point Likert scale. The questionnaire also included a Likert-type question about the user’s degree of agreement with the statement: “I am addicted to the Internet” and one question about gender and addiction: “Do you think that girls are more Internet-addicted than boys?”
- Addictive behaviors related to the Internet were assessed with the Cuestionario de Experiencias Relacionadas con Internet (CERI) [Questionnaire on Internet-related experiences] [18]. This questionnaire consisted of 10 items about Internet use that were answered on a four-point Likert scale. Item example: “Piensas que la vida sin Internet es aburrida, vacía y triste?” (Do you think that life without the Internet is boring, empty, and sad?). The reliability (Cronbach’s alpha) in the present study was 0.76; in the original study it was 0.77.
- Addictive behaviors related to the mobile phone were assessed with the Cuestionario de Experiencias Relacionadas al Móvil (CERM) (Questionnaire on experiences related to the mobile phone) (CERM) [18]. This questionnaire consisted of 10 items about mobile phone use that were answered on a four-point Likert scale. Item example: “Hasta qué punto te sientes inquieto cuando no recibes mensajes o llamadas?” (To what extent do you feel anxious when you do not receive messages or calls?). In the present study, Cronbach’s alpha was 0.73. Cronbach’s alpha in the original study was 0.80. Other studies have also reported reliability indexes of 0.80 [41].

For both CERI and CERM, the scores were calculated by adding up the answers to all the items, to a maximum of 40 points; cut-off points were established in a former study [42]. CERI and CERM have been used in several studies on adolescents’ excessive Internet and mobile phone use [19,43].

### 2.3. Procedure

Eligible participants were invited to participate in the present study by means of an email containing a link to a Google Docs form. No personal information was requested, and it was not possible to connect any of the data from the questionnaires to academic records. The participants had to click on a box to give their informed consent and continue with the study. The students did not receive any monetary or academic reward for their participation. The study was approved by the Committee of Ethics and Research of the FPCEE Blanquerna, Universitat Ramon Llull.

### 2.4. Data Analysis

Normality checks were run on the data. Student’s *t*-tests were run to assess gender differences in relation to: (a) the type of use they engage in on the Internet; (b) their scores on the CERI and CERM; (c) the degree of agreement that users expressed regarding the question “I’m addicted to the Internet.” To check if the use of certain Internet functions is associated with negative consequences of use, correlations were calculated between the use of the Internet functions and scores on the CERI and CERM. A multiple analysis of variance for gender and year of questionnaire administration was run to test the effects of these two factors on both the CERM and CERI scores.

## 3. Results

### 3.1. Results of the Present Study

The frequencies of Internet uses are shown in Table 1. The most frequent activities on the Internet were checking emails and sending messages, participating in social networks, and listening to music. The least frequent uses were gambling and visiting adult pages. Significant gender differences were found between all uses except for online purchases, viewing of TV series, movies or videos, and administrative tasks.

Descriptive statistics of the Cuestionario de Experiencias Relacionadas al Móvil (CERM) and Cuestionario de Experiencias Relacionadas con Internet (CERI) scores are presented in Table 2. The mean CERI score was 18.04 (SD = 4.50), and the mean CERM score was 15.77 (SD = 3.50) for the whole cohort 6. There were no significant differences either for gender or for the different major degrees (*F*(4, 787) = 1.24; *p* = 0.291 for the CERI, and *F*(4, 787) = 1.85; *p* = 0.116 for the CERM). To the question, “Do you think girls are more addicted to the Internet than boys?” 73.2% answered affirmatively.

The correlation between the CERI and the CERM was high (*r* = 0.76, *p* = 0.000). The correlations between the different online functions and the CERI and CERM were mostly significant, but low, with the most relevant correlation being between social networks and both the CERI and the CERM (see Table 3).

Regarding the self-assessment of whether they considered themselves addicted to the Internet, 375 students (47.4%) either agreed or agreed strongly with this statement. Table 4 shows that people who “strongly agreed” with the statement “I am addicted to the internet” obtained significantly higher results than the rest of the participants on both the CERI and the CERM. The correlations with the CERI and CERM were 0.38 (*p* < 0.001) and 0.34 (*p* < 0.001), respectively.

### 3.2. Comparison between Present Study and Former Studies with CERM and CERI

As shown in Table 5, the scores in the CERI and CERM grew from 2005 to 2013 and remained stable during the 2013–2014 academic year. The number of students who showed problematic Internet use went from 1.5% in 2005 to 6.4% in 2017 and from 0.6% to 3.0%, in the case of problematic mobile phone use. The correlation between CERI and CERM was “moderate” up to 2014 and increased to “high” as of 2015.

Descriptive statistics for the CERM and CERI scores, separately for males and females, over the period 2006–2017 are presented in Table 6. Cohorts 3, 4, and 5 were taken together, as data collection took place in the same year. The multivariate analysis of variance for the effects of gender and year of administration on CERI and CERM showed significant effects of both factors (except for gender on CERI) and also a combined effect (see Table 7).

**Figure 1:**
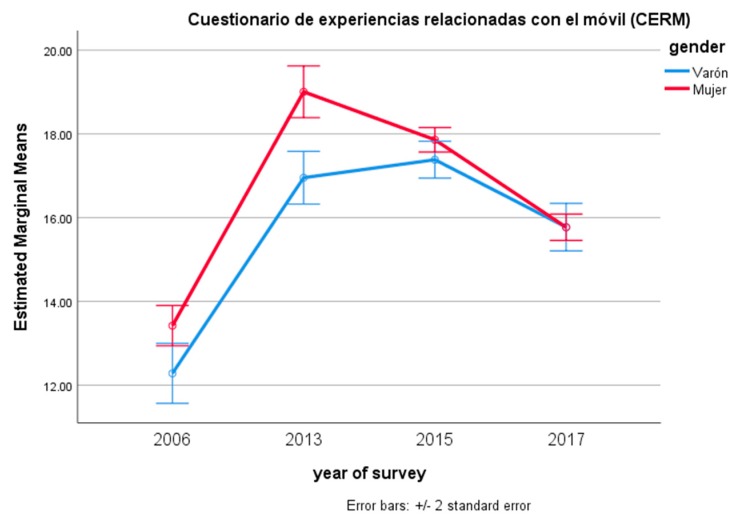
Estimated marginal means of Cuestionario de Experiencias Relacionadas al Móvil (CERM) for year of survey and gender.

**Figure 2:**
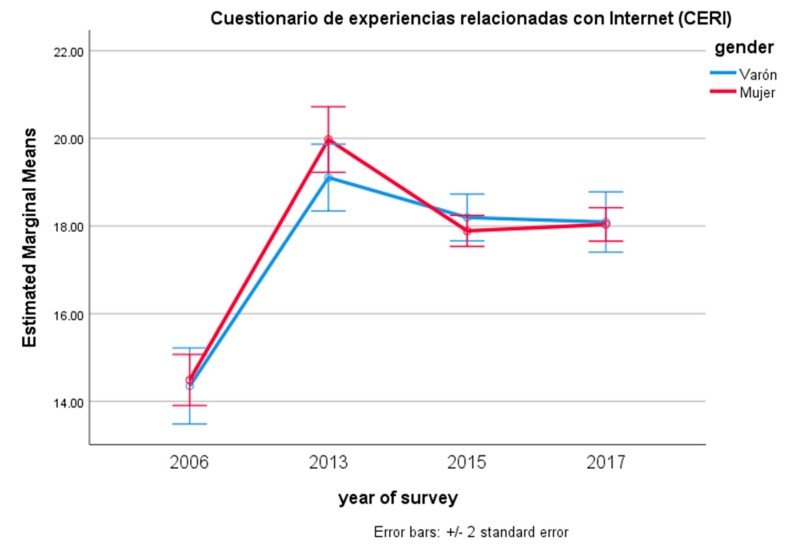
Estimated marginal means of the Cuestionario de Experiencias Relacionadas con Internet (CERI) for year of survey and gender.

Women presented higher scores in the CERM, but not in the CERI. Post-hoc Bonferroni pairwise comparisons showed that scores for both CERM and CERI had increased from the first survey: there was, in fact, a significant difference between the 2006 scores and those of all the following surveys (*p* < 0.001 in all comparisons). However, between the 2013 and the following years, there was no significant difference between 2013 and 2015, and there was even a decrease of the negative effects between 2015 and 2017 (*p* < 0.001) with respect to the CERM; for the CERI, there was a decrease from 2013 to 2015 and from 2015 to 2017 (*p* < 0.001 in both cases). Figure 1 and Figure 2 present the results separately for CERM and CERI and for both sexes. As can be seen, the increase of the perception of problematic Internet and smartphone use was stronger for females than for males, but as of 2017, both sexes had lower scores and tended to present the same degree of negative effects.

## 4. Discussion

The objective of this study was to evaluate the perception of problematic Internet and mobile phone use and compare these results with those of similar cohorts from up to a decade ago. One of the problems presented by studies evaluating technological addictions to the Internet, social networks, mobile phones, and video games is the absence of longitudinal studies. It is difficult for these studies to monitor a cohort in the medium or long term because the questionnaires are administered in person or online to: (i) cohorts obtained in the general population through social networks or the like; (ii) samples of high school or university students; (iii) video gamers identified in forums. Other factors that make longitudinal studies difficult are the need for respecting the participants’ anonymity and the existence of time limitations (i.e., on funding for the research projects and doctoral theses). To overcome these difficulties, this study compared data from different cohorts of university students assessed in different moments.

Our hypotheses were partially confirmed. Taken globally, the results of the study support the idea that the perception of a problematic use of the Internet and mobile phones exists and has increased over the last decade (H1). This perception seems to go along with the growth of Internet use and all kinds of electronic devices with a screen, with which our samples became familiar during their adolescence [44]. This problematic use of the Internet is specific and not general; that is, it depends on the concrete activity that is carried out [45,46,47]. This can also be affirmed for mobile phones [17,48,49]. Given that the Internet applications most widely used by university students are e-mail and messaging, participating in social networks, and listening to music, we infer that the increase in the perception of problematic use is associated with the use of online social networks. The activities in which the university students invest the least time are betting games and adult pages, like in other similar samples [17,45,46] and as is expected for university students. However, despite the existence of problematic use, it seems that the term ‘addiction’ is an inadequate construct when used as simply “Internet addiction”, because: (i) the problematic use does not depend on the mobile phone or the Internet itself, but on the activities accessed on them; (ii) the problematic use can be the symptom of other disorders, not a primary disorder in itself [10,17]; (iii) there is a risk that labeling this problematic use an addiction means pathologizing the daily life [50]. The questionnaires used make it possible to detect a concern about certain technology-based behaviors but in no case to issue a clinical diagnosis. The term ‘addiction’ is probably adequate when related to specific types of use, such as addiction to gaming via the Internet or to pornography via the Internet.

It is difficult to compare our data on problematic mobile use with those of other prevalence studies because of the use of different measuring instruments. A preliminary comparison shows that the range of values for problematic users or addicts ranges between 0% and 35%, with 10–20% being the most frequent values [7,10,12], although there have been reports of 48% in university students [24]. In a recent research, the percentages ranged from 3.9% in Belgium to 1% in Poland, with 1.7% addicts identified in the Spanish sample [17].

It is also not surprising that the Internet uses of young men and women are quite different from each other as there are differences in behavior and attitudes between them in the real world which are perpetuated in the network (H2). Our results show that women use social networks and academic applications more and listen to more music than men. Men play more videogames and betting games and use more adult pages than women. In any case, women’s problematic use is greater (H3), probably as a consequence of their using social networks more than males and of the role that those social networks play in communication and in creating and maintaining connections [17,40,51]. Some studies suggest that there may be different thresholds for males and females with respect to these negative effects [52].

The average scores in the CERI were higher than in the CERM, as has been the case with these instruments in other studies [18,20,53]. No differences were found between men and women in the problematic use of the mobile phone despite this being a frequent result in other investigations [15,17,40,53]. Although the Internet activities of men and women were different, there were no differences found in the problematic use of the Internet [53,54]. However, the perception of our students was that women are more addicted to the Internet than men probably because of the fact that using social networks is more common than using video games. Another possible explanation is that women are more vulnerable to this type of problem because it is related to communication practices such as establishing and actively maintaining relationships, which women engage in more than men [38,40].

Social network use is the only Internet use that moderately correlated with CERI and CERM. The other correlations, in line with other studies, were low or nonexistent [17]. In fact, online social networking is considered, along with video games, to be the use with the highest risk of becoming problematic [55,56] even though there is a lack of empirical confirmation [13]. Facebook and WhatsApp (as WhatsApp could be considered a social media) could be used as a key component to understand how young people socialize through these applications [57]. A large-scale tracking of online behavior showed that the use of WhatApp over the smartphone accounted for nearly 20% of all smartphone behavior, above Facebook use [58]. The low correlation with the different Internet uses can be explained because we are talking about a population that, as a whole, bets little and consumes little pornography, which leads us to think that we would obtain higher correlations if the CERI and the CERM were applied to cohorts of people extracted from the general population, who would be more likely to bet online, consume pornography, and/or be intensive video gamers.

The correlation between the CERI and CERM was high. In fact, we wonder if it is still convenient to use both the CERI and the CERM since, at the current time, both measures may be considered equivalent in the context of young people’s technology use; young people use the mobile phone more and more frequently to access the Internet [8,17,40] and tend not to distinguish between the platforms (mobile vs. computer) and the program/application. The current study confirms how strong smartphone and Internet addiction and social media overlap [47,59] and advises that the evolution of technology forces us to change and update certain research questions. For example, when we designed the CERM, the mobile phones used by the participants did not have access to the Internet, whereas at present, there is not really a distinction between mobile phones and smartphones because they are considered synonymous. We will draw upon an anecdote to illustrate this situation. When, over a decade ago, the first papers expressing concern about mobile phone addiction were published [9], they were about mobile phones without Internet access. However, last year, when we showed a fourth-year psychology student a picture of an old Nokia phone from that era, one of them asked: “But... was it possible to be addicted to that?” This question reveals to what extent the diagnosis of technological addictions is influenced by time and social and cultural change.

From our point of view, the concern over mobile phone addiction came in two distinct waves. The first was focused on the non-smart mobile phone and was mainly due to two factors: the amount of phone bills that the use led to and the high use of text messages. Phone bills were a point of concern because a flat rate did not exist, and users needed a certain learning period to understand how to manage their use so that it remained within reasonable limits. Another point of concern was the number of text messages (i.e., for women: 11 or more calls or text messages per day, see Thomee, Dellve, Harenstam, Hagberg [6]). Several studies can serve as examples [6,9,34,36]. When, thanks to new billing structures, users managed to control their phone expenses and these worries seemed to dissipate, smartphones emerged on the market. At that point, a new wave of concern started in regard to this new device, because it allowed access to the Internet and Internet-based applications such as social networks and messaging services. Several studies can serve as examples [22,23,24,60,61]. Here again, the influence of context and culture is crucial.

Something similar is occurring with Internet addiction. In the last decade, we have learned that it is convenient to distinguish between behavioral addictions that take place on the Internet (for example, pathological gambling), the specific uses of the Internet that can become problematic (for example, videogames and social networks), and a possible generalized Internet addiction [45,46,49]. Davis [62] offered one of the first theoretical models, which differentiates between a generalized and a specific type of Internet addiction. Later, more sophisticated models to explain the different levels of the addiction process have been developed, such as the I-PACE model [27], which is useful for understanding the development and maintenance of specific Internet-use disorders. The students who responded to the CERI 10 years ago did so thinking primarily about their connection to the Internet via their computer, whereas now they access the Internet indiscriminately from their mobile phones, other handheld devices, and home or university computer. One example of how Internet use has changed in recent years is the application WhatsApp. WhatsApp is a telephone messaging service but it shares many features with social networks and, as of 2016, it can be accessed from the computer. Therefore, students can use WhatsApp on their mobile phones or from their laptop when they are in the classroom and they create class groups on both WhatsApp and Facebook indiscriminately. Another example of the merging of lines between phone applications and Internet applications is the difficulty in distinguishing between how much time is dedicated to each application or program, since it is so common to work in multi-screen mode. Students can write an academic paper, answer emails, and have a conversation on WhatsApp, all at the same time.

The results of the comparison between the present and former studies with the CERM and the CERI show that the negative effects of Internet and mobile phone use are considerably stronger now that at the first survey in 2006. However, this is due mainly to the first period, between 2006 and 2013. Apparently, there is a downward tendency in the perception of negative consequences in the recent years, which may correspond to a progressive normalization and integration of these new technologies into our daily life.

Although we have already mentioned the limitations of the CERI and CERM (they were created in the cell phone era before the existence of the smartphone and they are self-report measures), they are easy to use and to score, thereby inviting us to continue using them when possible in order to study the evolution of the perception of the problematic use of the Internet and mobile phones. They indicate, not so much the prevalence of an addiction, but the perception of a problem by the respondents. Although the correlation of the CERI and CERM with the statement “I am addicted to the internet” was moderate, the participants who were “very much in agreement” and “agreed” with this statement obtained significantly higher results than the rest in both the CERI and the CERM. This indicates that it might be possible to use this single question to detect the perception of problematic mobile phone use of individuals, as has already been suggested [46,63]. It is highlighted that our students seem to have a perception about their Internet addiction more in line with the data obtained through the questionnaires than the participants in the study done by Pontes, Szabo, and Griffiths [46], in which 51.9% of the participants identified themselves as Internet addicts.

This study is not without limitations. Firstly, university students have a higher than average level of academic development and their use of the Internet and mobile phones is not necessarily representative of the use that other young people engage in. Secondly, a problematic use of these technologies does not correspond to any diagnostic entity and may be a reflection of their social impact. Thirdly, both the CERI and the CERM should be updated, like other mobile addiction instruments (see, Kuss, Harkin, Kanjo and Billieux [64]), because a general use of the Internet is no longer conceivable; rather, a specific one is, and more so because of the new and expanded uses of smartphones. The terms “internet addiction” and “smartphone addiction” could be ‘misnomers’ [49] which we might not want to use anymore. Finally, it is possible that some differences found in this study were more influenced by the cultural differences between the samples than by the temporal differences.

## 5. Conclusions

Although men and women use the Internet differently, their problematic use of the Internet and mobile phones are quite similar. In university students, the use of social networks is the main factor responsible for the perception of problematic use; a casuistry that has increased in the decade 2006–2016. Despite the limitations of the CERI and CERM, estimating the prevalence at different points in time offers valuable information about the evolution of the perception of problematic Internet and mobile phone use. It is convenient to repeat the studies using the same instrument in order to understand the perception of problematic Internet and mobile phone use even if it lacks clinical significance. The degree of agreement with the statement “I am addicted to the Internet” might be used as a screening question for the problematic use. Young people are worried about the phenomenon, and it is convenient to keep track of their perception on the issue in order to design, if necessary, educational campaigns for an adequate use of these technologies.

## Figures and Tables

**Table 1:** Most frequent Internet uses by university students.

Internet Uses	Men	Women	Total	*t*	*p*
	*M* (SD)	*M* (SD)	*M* (SD)		
Phone calls and videoconferences	2.70 (1.12)	2.98 (1.11)	2.91 (1.27)	2.97	0.003
Email/Chat	4.60 (0.66)	4.76 (0.54)	4.72 (0.58)	3.38	0.001
Social networking	4.11 (1.06)	4.34 (0.97)	4.28 (0.99)	2.69	0.007
General information	3.80 (1.04)	3.33 (1.07)	3.44 (1.08)	5.18	0.007
Shopping	2.11 (1.00)	2.11 (1.06)	2.11 (1.05)	0.01	0.987
Videogames	2.32 (1.29)	1.63 (0.93)	1.79 (1.07)	7.94	0.000
Gambling/betting	1.44 (0.85)	1.06 (0.32)	1.15 (0.53)	8.96	0.000
Videos/TV series	3.43 (1.24)	3.52 (1.21)	3.50 (1.22)	0.90	0.364
Listening to music	3.99 (1.10)	4.24 (1.01)	4.18 (1.04)	2.90	0.004
Administrative tasks	2.58 (1.20)	2.60 (1.25)	2.59 (1.24)	0.21	0.832
Adult content	2.34 (1.08)	1.25 (0.65)	1.51 (0.90)	16.07	0.000
Academic activities	3.66 (1.01)	4.03 (0.99)	3.95 (1.01)	4.46	0.000

**Table 2:** Means and standard deviations of the Cuestionario de Experiencias Relacionadas con Internet (CERI) and the Cuestionario de Experiencias Relacionadas al Móvil (CERM) scores for cohort 6.

Questionnaire	Men	Women	Total	*t*-Tests
	*M* (SD)	*M* (SD)	*M* (SD)	
Cuestionario de Experiencias Relacionadas al Móvil (CERM)	15.77 (3.55)	15.77 (3.50)	15.77 (3.50)	*t*(790) = 0.012 (*p* = 0.884)
	*n* = 186	*n* = 606	*n* = 792	
Cuestionario de Experiencias Relacionadas con Internet (CERI)	18.09 (4.81)	18.04 (4.41)	18.04 (4.50)	*t*(790) = 0.146 (*p* = 0.990)
	*n* = 186	*n* = 606	*n* = 792	

**Table 3:** Correlations between CERI or CERM and the uses of the Internet.

Internet Uses	CERI	CERM
Email/Chat	0.15 **	0.14 **
Social networking	0.23 **	0.21 **
General information	0.14 **	0.08
Shopping	0.14 **	0.12 **
Videogames	0.10 **	0.11 **
Gambling/betting	0.15 **	0.17 **
Videos/TV series	0.12 **	0.11 **
Listen music	0.18 **	0.17 **
Administration	0.03	0.04
Adult content	0.12	0.13 **
Academic activities	0.07	0.01
Phone calls/videoconferences	0.04	0.08

Note: ** *p* < 0.001.

**Table 4:** Levels of agreement with the statement “I am addicted to the Internet” with the scores in CERI and CERM.

Level of Agreement	CERI	CERM
	*M* (SD)	*M* (SD)
Strongly agree	21.89 (4.71)	18.47 (4.22)
	*n* = 93	*n* = 93
Agree	19.02 (4.20)	16.45 (3.25)
	*n* = 282	*n* = 282
Neither agree nor disagree	16.82 (3.81)	14.89 (3.09)
	*n* = 275	*n* = 275
Disagree	15.90 (3.58)	14.35 (2.71)
	*n* = 123	*n* = 123
Totally disagree	16.47 (6.85)	14.47 (4.25)
	*n* = 19	*n* = 19

**Table 5:** Scores of the university student cohorts in the CERI and the CERM.

Cohort	Year of Survey	CERI	CERM	Correlation CERI/CERM	Problematic Use CERI (%)	Problematic Use CERM (%)
		*M* (SD)	*M* (SD)			
Cohort 1	2006	14.44 (4.00)	13.07 (2.90)	0.439 **	2.2%	0.9%
Cohort 2	2013	19.65 (5.06)	17.83 (4.39)	0.530 **	9.2%	8.6%
Cohort 3	2015	18.64 (5.03)	18.38 (4.09)	0.692 **	7.5%	8.1%
Cohort 4	2015	17.05 (4.06)	16.68 (3.51)	0.734 **	2.0%	3.0%
Cohort 5	2015	17.98 (5.41)	17.88 (4.98)	0.851 **	9.3%	11.5%
Cohort 6	2017	18.04 (4.50)	15.77 (3.50)	0.760 **	6.4%	3.0%

Note: ** *p* < 0.001.

**Table 6:** Means and standard deviations (in brackets) of the CERM and the CERI scores.

Questionnaire	2006		2013		2015		2017	
	Males	Females	Males	Females	Males	Females	Males	Females
CERM	12.28 (2.62)	13.42 (2.96)	16.95 (4.34)	19.01 (4.60)	17.39 (4.40)	17.86 (4.18)	15.77 (3.55)	15.77 (3.51)
CERI	14.35 (4.05)	14.49 (3.99)	19.11 (4.97)	19.97 (5.20)	18.19 (5.11)	17.89 (4.85)	18.09 (4.81)	18.04 (4.41)

**Table 7:** Tests of between-subjects effects for the factors gender and year in CERM and CERI.

Source	Variable	*F*	*p*	η^2^
gender	CERM	24.16	<0.001	0.010
	CERI	0.508	0.476	0.000
year	CERM	140.99	<0.001	0.146
	CERI	70.99	<0.001	0.079
Gender × year	CERM	5.29	0.001	0.006
	CERI	1.21	0.304	0.001
